# miR-182 Modulates Myocardial Hypertrophic Response Induced by Angiogenesis in Heart

**DOI:** 10.1038/srep21228

**Published:** 2016-02-18

**Authors:** Na Li, Cheol Hwangbo, Irina M. Jaba, Jiasheng Zhang, Irinna Papangeli, Jinah Han, Nicole Mikush, Bruno Larrivée, Anne Eichmann, Hyung J. Chun, Lawrence H. Young, Daniela Tirziu

**Affiliations:** 1 Yale Cardiovascular Research Center, Section of Cardiovascular Medicine, Department of Internal Medicine, Yale School of Medicine, New Haven, CT, 06520, USA; 2 Paris Cardiovascular Research Center PARCC, Inserm U970, 56 Rue Leblanc, 75015 Paris, France; 3 Department of Molecular Physiology, Yale University School of Medicine, New Haven, CT, 06520, USA

## Abstract

Myocardial hypertrophy is an adaptive response to hemodynamic demands. Although angiogenesis is critical to support the increase in heart mass with matching blood supply, it may also promote a hypertrophic response. Previously, we showed that cardiac angiogenesis induced by placental growth factor (PlGF), promotes myocardial hypertrophy through the paracrine action of endothelium-derived NO, which triggers the degradation of regulator of G protein signaling 4 (RGS4) to activate the Akt/mTORC1 pathways in cardiomyocytes. Here, we investigated whether miRNAs contribute to the development of hypertrophic response associated with myocardial angiogenesis. We show that miR-182 is upregulated concurrently with the development of hypertrophy in PlGF mice, but not when hypertrophy was blocked by concomitant expression of PlGF and RGS4, or by PlGF expression in eNOS^−/−^ mice. Anti-miR-182 treatment inhibits the hypertrophic response and prevents the Akt/mTORC1 activation in PlGF mice and NO-treated cardiomyocytes. miR-182 reduces the expression of Bcat2, Foxo3 and Adcy6 to regulate the hypertrophic response in PlGF mice. Particularly, depletion of Bcat2, identified as a new miR-182 target, promotes Akt^Ser473^/p70-S6K^Thr389^ phosphorylation and cardiomyocyte hypertrophy. LV pressure overload did not upregulate miR-182. Thus, miR-182 is a novel target of endothelial-cardiomyocyte crosstalk and plays an important role in the angiogenesis induced-hypertrophic response.

Cardiac hypertrophy is a compensatory adaptation to increased hemodynamic demands. While in normal conditions the coronary vasculature provides sufficient oxygen and nutrient delivery, during myocardial hypertrophy an increase in coronary blood supply is critical to support the increase in heart mass and function^1^. Although cardiomyocytes secrete growth factors that can induce an angiogenic response, in some cases the extent of angiogenesis may be limited and imbalanced relative to the extent of hypertrophy, which ultimately leads to heart failure^2^,^3^,^4^,^5^. On the other hand, several studies collectively demonstrate that angiogenesis may conversely promote myocardial hypertrophy^6^,^7^,^8^,^9^,^10^,^11^,^12^.

Previously, we showed that cardiac-specific transgenic expression of angiogenic factors PR39^7^ or placental growth factor (PlGF)^11^ promotes myocardial hypertrophy through a paracrine mechanism mediated by the endothelium-released nitric oxide (NO) that coordinates angiogenesis and cardiomyocyte growth. Our studies focused on the role of NO in the degradation of regulator of G protein signaling type 4 (RGS4) through the Arg/N-end rule pathway of protein degradation^13^. RGS4 is a GTPase-activating protein for heterotrimeric Gq and Gi, which are associated with the hypertrophic response in failing human myocardium^14^. We determined that the increased vascular density and higher baseline NO production accelerated the degradation of RGS4 and promoted cardiomyocyte hypertrophy by relieving the repression of Gβγ-dependent PI3Kγ activation of the Akt/ mTORC1 pathway^11^. Moreover, the angiogenesis-induced myocardial hypertrophy was characterized by normal LV contractile function and no evidence of fibrosis or induction of markers associated with pathological hypertrophy^11^. Additional factors might modulate the activation of the Akt/mTORC1 pathway and we therefore investigated whether miRNAs contribute to the induction of the hypertrophic response associated with myocardial angiogenesis.

Thus, we studied the expression pattern of miRNAs in the mouse model of PlGF-induced myocardial angiogenesis^11^ and the effects of anti-miRs in these mice. We also assessed whether NO and RGS4 were critical for the regulation of miRNA identified during the hypertrophic response using mouse models and cultured cardiomyocytes. Furthermore, we assessed whether miRNA involved in the angiogenesis-induced hypertrophic response was also associated with the pathological response to LV pressure overload induced by transverse aortic constriction (TAC). Our findings show that upregulation of miR-182 is associated with angiogenesis-induced myocardial hypertrophy and decreases the expression of branched chain aminotransferase 2 (Bcat2), forkhead box O3 (Foxo3), and adenylate cyclase type 6 (Adcy6). We also found that newly identified miR-182 target Bcat2, a critical enzyme responsible for branched chain amino acids (BCAAs) catabolism, plays an important role in the activation of the Akt/mTORC1 pathway and the modulation of hypertrophic response in cardiomyocytes.

## Results

### miR-182 is upregulated subsequent to angiogenic stimulation and is dependent on NO-induced RGS4 degradation

**Figure 1:**
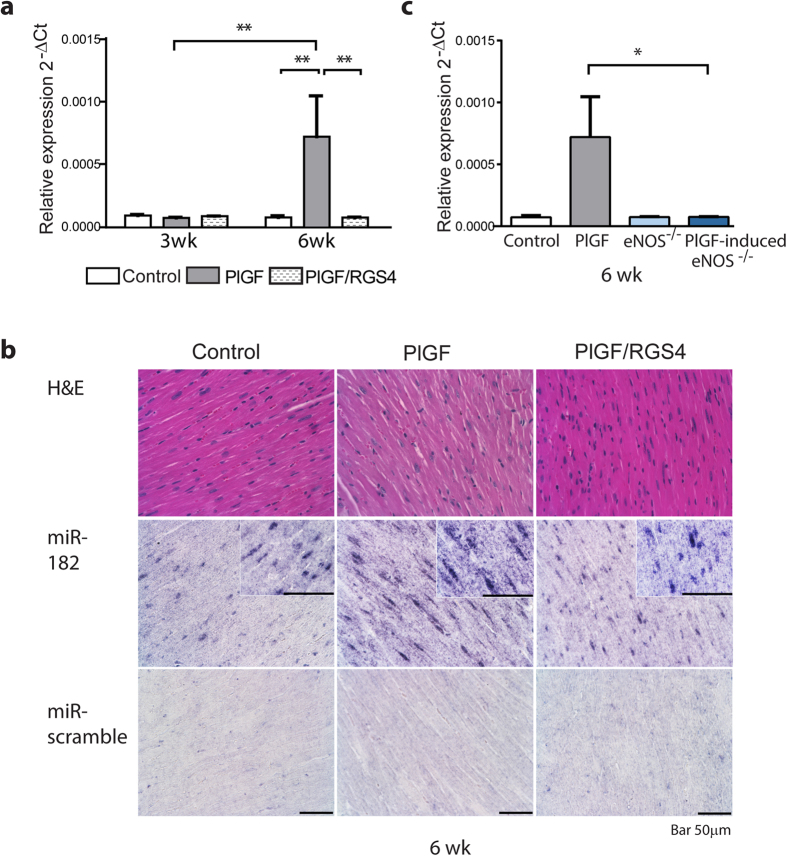
miR-182 upregulation in the heart subsequent to PlGF-induced angiogenesis is dependent on NO-induced RGS4 degradation mechanism. (**a**) Increased miR-182 expression in PlGF mice, after 6 weeks of angiogenic stimulation, is concurrent with the development of hypertrophy. miR-182 is not induced in PlGF/RGS4 mice, where hypertrophic response is inhibited. n = 6 (3 wk controls); 6 (3wk PlGF); 6 (3 wk PlGF/RGS4); 6 (6 wk controls); 5 (6 wk PlGF); 6 (6 wk PlGF/RGS4). (**b**) *In situ* hybridization of LV myocardium sections with DIG labeled miRCURY LNA mmu-miR-182 detection probe or DIG labeled miR-scramble probe. (**c**) miR-182 is not upregulated in PlGF-induced eNOS^−/−^ mice, where the hypertrophic response is inhibited. n = 6 (controls); 5 (PlGF); 5 (eNOS^−/−^); 8 (PlGF/eNOS^−/−^). *P <  0.05; **P < 0.01.

To investigate whether miRNAs are involved in the regulation of the hypertrophic response subsequent to angiogenic stimulation, we employed a cardiac specific inducible Tet-OFF mouse model of PlGF expression driven by tTA under the αMHC promoter^11^. In this model, we have previously shown that myocardial hypertrophy develops subsequent to angiogenesis and requires 6 weeks to occur^11^ (summarized in Supplementary Fig. S1 on line). Microarray profiling of miRNAs in LV myocardium in PlGF mice after 3 and 6 weeks of angiogenic stimulation revealed the upregulation of miR-182 expression at 6 weeks (Supplementary Fig. S1 on line). Furthermore, qPCR analysis confirmed the specific increase of miR-182 expression (by ~9-fold) in PlGF mice at 6 weeks concurrent with the development of hypertrophy, but not at 3 weeks when only angiogenesis was apparent (Fig. 1a).

To further investigate the role of miR-182 in angiogenesis-induced cardiac hypertrophy, we determined its expression in the PlGF/RGS4 mouse, where induced cardiac expression of RGS4 concurrently with PlGF inhibits the hypertrophic response^11^. In contrast to the PlGF mice, miR-182 expression in PlGF/RGS4 mice was low and similar to control mice (Fig. 1a). Consistent with the quantitative PCR data in heart homogenates, *in situ* hybridization of miR-182 detected higher expression of miR-182 within the ventricular myocardium in PlGF mice, compared with PlGF/RGS4 mice and controls (Fig. 1b). Furthermore, the co-staining of miR-182 with cardiomyocyte marker, cardiac troponin T confirmed the presence of miR-182 in cardiomyocytes (Supplementary Fig. S2 on line). These data indicate that both the upregulation of miR-182 and the hypertrophic response are prevented when RGS4 expression is maintained.

Myocardial hypertrophy subsequent to angiogenesis requires endothelial-NO production to induce RGS4 degradation^11^, and thus we investigated whether miR-182 upregulation in PlGF mice was NO dependent by inducing PlGF expression in eNOS^−/−^ mice. Interestingly, consistent with the inhibition of hypertrophic response^11^, we found abrogation of miR-182 upregulation in PlGF-induced eNOS^−/−^ mice (Fig. 1c). Together these data indicate that miR-182 upregulation subsequent to PlGF-induced angiogenesis is dependent on NO-induced RGS4 degradation mechanism, whereas PlGF has no direct effect on miR-182 regulation.

### Inhibition of miR-182 prevents NO-induced cardiomyocyte hypertrophy *in vitro*

**Figure 2:**
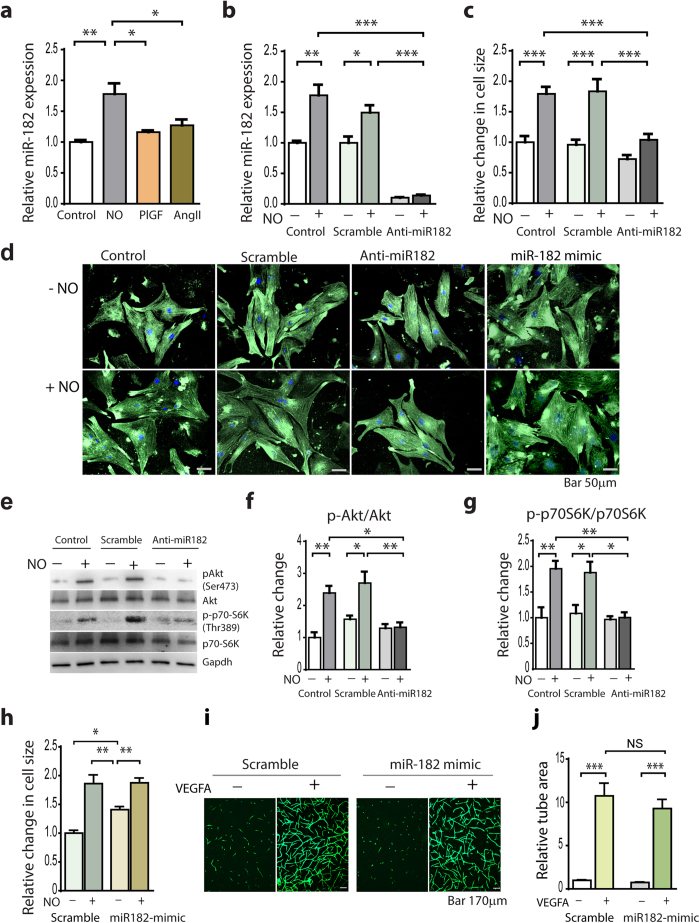
Inhibition of miR-182 prevents NO-induced cardiomyocyte hypertrophy. (**a**) miR-182 is upregulated in NRCs treated for 24 hrs with NO donor (DETA-NONOate, 100 μM), but not in PlGF (50 ng/ml) or Ang II (0.5 μM) treated NRCs. (**b**) miR-182 inhibition with anti-miR-182 (50 nM) in NO-treated NRCs, compared with miR-scramble (50 nM). (**c**) Anti-miR-182 prevents the increase in cell size in NO-treated NRCs. (**d**) Representative NRCs transfected with anti-miR-182, miR-182 mimic and miR-scramble with and without NO donor treatment for 24 hrs compared with control NRCs (co-staining with an anti-α-actinin sarcomeric Ab and DAPI). (**e**) Western blot analysis of Akt^Ser473^ and p70-S6K^Thr389^. (**f**) Relative change in Akt activation at Akt^Ser47^. (**g**) Relative change in p70S6K activation at p70-S6K^Thr389^. (**h**) miR-182 mimic promotes NRC hypertrophy. (**i**) Representative HUVECs sprouting (calcein AM staining) after transfection with miR-182 mimic or miR-scramble (50 nM). (**j**) Quantitative analysis of the tube area. * *P *<  0.05; **P < 0.01; ***P < 0.001.

Next, we determined whether NO can directly induce miR-182 expression in cardiomyocytes by treating neonatal rat cardiomyocytes (NRCs) with the NO donor (DETA-NONOate). We found significant upregulation of miR-182 expression in response to NO donor (Fig. 2a). In contrast, the treatment of NRCs with PlGF, or the cardiac hypertrophic GPCR agonist Ang II did not affect miR-182 expression (Fig. 2a). In order to assess whether miR-182 plays a role in NO-induced cardiomyocyte hypertrophy we inhibited miR-182 in NO-treated NRCs with antagomir. As shown in Fig. 2b, the treatment with anti-miR-182 antagonized miR-182 expression and attenuated the hypertrophic response induced by the treatment with NO donor (Fig. 2c,d). Because NO treatment activates Akt^Ser473^ and downstream mTORC1 target p70-S6K^Thr389 ^11, we tested whether the inhibition of miR-182 blocked the activation of Akt/mTORC1 pathway in NO-treated NRCs. In agreement with the inhibition of the hypertrophic response, anti-miR-182 also diminished the phosphorylation of Akt^Ser473^ and p70-S6K^Thr389^ in NO-treated NRCs (Fig. 2e–g).

To assess whether miR-182 has a direct hypertrophic effect, we treated NRCs with a miR-182 mimic. While a 24-hrs treatment with NO donor increased cell size by 85%, the 24-hrs treatment with miR-182 mimic increased the cell size by 40%, compared with miR-scramble treatment (Fig. 2d,h). The treatment of NRCs with miR-182 and NO donor did not stimulate the cell growth further than the effect observed with NO donor alone (Fig. 2h). Consistent with the pro-hypertrophic effect in NRCs, miR-182 induced a significant increase in p70-S6K^Thr389^ phosphorylation (Supplementary Fig. S3 on line). These data in conjunction with the more dramatic reciprocal effects of anti-miR-182 treatment in NO-treated NRCs indicate that the activation of the Akt/mTORC1 pathway downstream of NO initiates the hypertrophic response, whereas miR-182 functions as a modulator.

Finally, to determine whether miR-182 has any effect on angiogenesis, we examined endothelial cell sprouting *in vitro*. Transfection of HUVECs with miR-182 mimic did not affect endothelial cell sprouting in response to VEGFA, as compared with miR-scramble, suggesting no direct effect of miR-182 on angiogenesis (Fig. 2i,j).

### Inhibition of miR-182 prevents the hypertrophic response subsequent to angiogenesis in heart

We then proceeded to study whether miR-182 has an important role in modulating the hypertrophic response *in vivo*. PlGF mice were treated with intravenous injection of anti-miR-182 or control miR-scramble during the hypertrophic phase (the last 3 weeks of the 6 week angiogenic stimulation period) (Supplementary Fig. S4 on line). Treatment with anti-miR-182 reduced the cardiac content of miR-182 in PlGF mice by 50%, compared with control miR-scramble treatment (Supplementary Fig. S4 on line). Anti-miR-182 treatment led to a 40% reduction (p = 0.057) in liver, whereas in kidney miR-182 content was not affected (Supplementary Fig. S4 on line).

**Figure 3:**
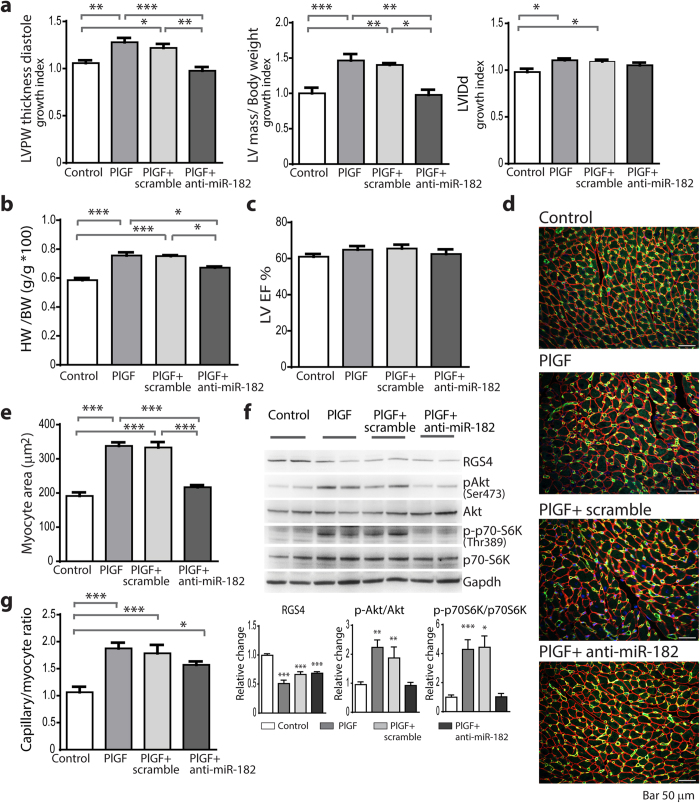
Inhibition of miR-182 prevents myocardial hypertrophy and Akt/mTORC1 activation. (**a**) Growth index of echo-determined: LV posterior wall (LVPW) thickness, LV mass (normalized to body weight) and LV internal diameter in diastole (LVIDd) in untreated PlGF mice and PlGF mice treated with either anti-miR-182 or miR-scramble, compared with control. (**b**) Heart weight (HW)/body weight (BW) ratio at the end of treatment. (**c**) Echo-determined LV ejection fraction (LVEF). (**d**) Representative LV myocardium sections immunostained with anti CD31 and anti-laminin Abs. (**e**) Cardiomyocyte cross-sectional area measurements. (**f**) Western blot analysis of RGS4, Akt^Ser473^, p70-S6K^Thr389^ expression in LV tissue lysates. (**g**) Capillary/myocyte ratio. n = 9 (controls); 9 (PlGF); 6 (PlGF+miR-scramble); 6 (PlGF+anti-miR-182). *P < 0.05; **P < 0.01; ***P < 0.001 vs. control or as indicated.

The degree of LV hypertrophy was measured by echocardiography and calculated as a growth index that represents the ratio of parameters after treatment to the measurements prior treatment (Supplementary Fig. S4 on line). There was a significant increase of LV posterior wall (LVPW) thickness in PlGF mice, which was blocked by anti-miR-182 treatment, but not by control miR-scramble treatment (Fig. 3a). Similarly, in PlGF mice there was a 40% increase in LV mass, which was prevented by the treatment with anti-miR-182, but not by miR-scramble (Fig. 3a). Furthermore, the minor LV dilation observed in PlGF mice was also prevented by the treatment with anti-miR-182 (Fig. 3a). Consistent with the echocardiographic parameters, anti-miR-182 treatment also inhibited the increase in the heart weight to body weight ratio (HW/BW) in PlGF mice (Fig. 3b). Moreover, there was no impairment of LV contractile function in PlGF mice, and LV ejection fraction (EF) was also unaffected by the treatment with anti-miR-182 (Fig. 3c). These results indicate that miR-182 has an important role in the hypertrophic response induced by angiogenesis in heart.

To more directly assess the effect of anti-miR-182 on cardiomyocyte hypertrophy, we measured cardiomyocyte cross-sectional area in LV sections from PlGF mice with and without anti-miR-182 treatment. The results showed a significant inhibition of cardiomyocyte hypertrophy in anti-miR-182-treated PlGF mice, compared with either untreated or miR-scramble treated PlGF mice (Fig. 3d,e).

In PlGF mice, the NO-mediated RGS4 degradation releases the inhibition of Akt/mTORC1 pathway and induces an increase in Akt^Ser473^ and mTORC1/p70-S6K^Thr389 ^11. In contrast to PlGF and miR-scramble-treated PlGF mice that had increased phosphorylation at Akt^Ser473^ (2-fold) and 70-S6K^Thr389^ (4-fold), the levels of phosphorylated Akt^Ser473^ and p70-S6K^Thr389^ in anti-miR-182-treated PlGF mice were not increased and were comparable with controls (Fig. 3f). Thus, these *in vivo* results are consistent with the *in vitro* data showing an inhibitory effect of anti-miR-182 on Akt^Ser473^ and p70-S6K^Thr389^ levels and hypertrophy in NRCs (Fig. 2c–g). Importantly, the RGS4 protein levels, compared with controls, were similarly decreased regardless of whether PlGF mice were treated with or without anti-miR-182 (Fig. 3f). Also, the treatment with anti-miR-182 did not affect PlGF-induced angiogenesis, based on the findings that the increase in vessel density was sustained during either control miR-scramble or anti-miR-182 treatment (Fig. 3g). Thus, taken together, these results indicate an important regulatory effect of miR-182 on the Akt/mTORC1 pathway that is independent of NO-induced RGS4 degradation.

### miR-182 targets Bcat2, Foxo3, and Adcy6 to potentially regulate the hypertrophic response

**Figure 4:**
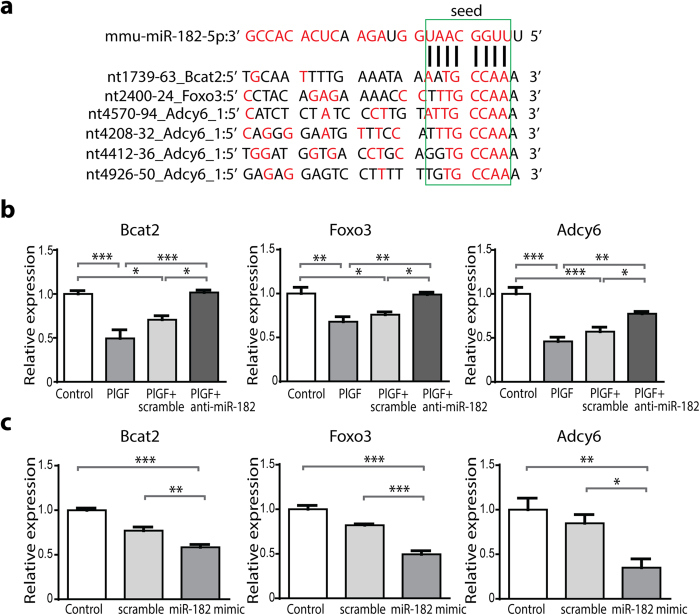
miR-182 downregulates Bcat2, Foxo3 and Adcy6 in PlGF mice and MEFs. (**a**) Alignment of mmu-miR-182 with putative 3′UTR target sites: one site in Bcat2 and Foxo3 and 4 sites in Adcy6. miR-182 seed sequence and the corresponding target sites are indicated in green box. Complementary bases are shown in red color. (**b**) Bcat2, Foxo3 and Adcy6 transcripts are downregulated in PlGF mice and control miR-scramble-treated PlGF mice, compared with controls. Anti-miR-182 treatment completely restored Bcat2 and Foxo3 expression and partially restored Adcy6 expression in PlGF mice. n = 4 (controls); 4 (PlGF); 4 (PlGF + control miR-scramble); 5 (PlGF + anti-miR-182). (**c**) Reduction of Bcat2, Foxo3 and Adcy6 expression in MEFs transfected with miR-182 mimic (50 nM). n = 4 independent experiments. *P < 0.05; **P < 0.01; ***P < 0.001.

To better understand the regulation of the Akt/mTORC1 pathway and to identify the miR-182 targeted genes, which might be linked to the hypertrophic response subsequent to angiogenesis, we conducted comparative mRNA expression profiling in PlGF mice (angiogenesis with hypertrophic response) and PlGF/RGS4 mice (angiogenesis with inhibition of hypertrophic response). The mRNA transcripts that were selectively downregulated on microarrays of PlGF mouse hearts, but not of PlGF/RGS4 mouse hearts, were further analyzed using the miRWalk database of predicted and validated microRNA targets^15^. Using this approach, we identified 9 potential miR-182 targets associated with the hypertrophic response in PlGF mice (Supplementary Table S1 on line). Of these targets, 3 miR-182 targets: Bcat2, Foxo3 and Adcy6 were further validated by qPCR analysis (Fig. 4a).

Coincident with the upregulation of miR-182 expression in PlGF mice, there was significantly reduced expression of Bcat2, Foxo3 and Adcy6 transcripts (Fig. 4b). Consequently, the treatment with anti-miR-182 partially restored the expression of Adcy6 and completely restored the expression of Bcat2 and Foxo3 to control levels (Fig. 4b).

Next, we addressed whether miR-182 negatively regulated the expression of Bcat2, Foxo3 and Adcy6 transcripts in an isolated cell system. The treatment of MEFs with a miR-182 mimic resulted in a ~65% reduction of Adcy6 and a ~45% reduction of Bcat2 and Foxo3 expression (Fig. 4c), consistent with our *in vivo* observations.

### Angiogenesis-induced hypertrophic response augments myocardial hypertrophy during LV pressure overload and protects against early contractile dysfunction

**Figure 5:**
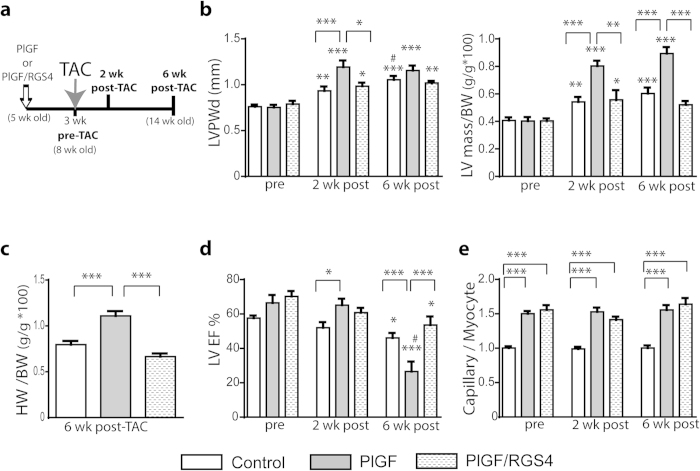
Angiogenesis-induced hypertrophic response augments myocardial hypertrophy during LV pressure overload. (**a**) PlGF and PlGF/RGS4 mice were subjected to TAC after 3 weeks of angiogenesis stimulation. All groups were examined at 2 and 6 weeks after TAC. (**b**) Echo-determined LVPWd thickness and LV mass/BW ratio at 2 and 6 weeks after TAC. Greater myocardial hypertrophy in PlGF mice after TAC, compared with PlGF/RGS4 and control mice. (**c**) HW/BW ratio 6 weeks after TAC in all groups. (**d**) Echo-determined LVEF prior and at 2 and 6 weeks after TAC. (**e**) Capillary/myocyte ratio in all groups prior to TAC and up to 6 weeks after TAC. n = 11 (controls); 6 (PlGF); 5 (PlGF/RGS4). *P < 0.05, **P < 0.01, ***P < 0.001 vs. pre-TAC or as indicated; # *P *< 0.05 vs. 2 wk post-TAC.

In order to determine whether hypertrophy induced by angiogenic stimulation has a molecular signature distinct from the pathological hypertrophic response to LV pressure overload and/or has a beneficial effect, PlGF and PlGF/RGS4 mice were subjected to TAC after angiogenic stimulation for 3 weeks (schematically described in Fig. 5a). After 3 weeks of angiogenesis, there was no cardiac hypertrophy at the time of TAC in PlGF or PlGF/RGS4 mice (Fig. 5b), consistent with our prior data^11^. As expected, at 2 and 6 weeks post-TAC, echocardiography demonstrated significant hypertrophy in all groups. However, the increase in LVPW thickness and LV mass was ~2-fold higher in PlGF mice than in either control or PlGF/RGS4 mice (Fig. 5b). A greater degree of myocardial hypertrophy in PlGF mice was also evidenced by higher HW/BW ratios (Fig. 5c). Although in PlGF mice the heart doubled in size by 2 weeks after TAC, the cardiac contractile function was preserved with significantly higher LVEF than in control mice subjected to TAC (Fig. 5d). However, there was a subsequent decline in cardiac function in all groups 6 weeks after TAC, with the PlGF mice having an even lower LVEF than control or PlGF/RGS4 mice (LVEF: 27% in PlGF mice vs. 46% in controls and 54% in PlGF/RGS4 mice, p* *< 0.001) (Fig. 5d). In PlGF/RGS4 mice, the angiogenesis-induced hypertrophic response was completely prevented compared with PlGF mice and the extent of myocardial hypertrophy and the decline in cardiac function after TAC were each comparable with control (Fig. 5b–d).

To exclude the possibility that the severe compromise in contractile function after TAC in PlGF mice could be due to a failure of angiogenesis during pressure overload we assessed the capillary density in LV sections. However, we found that the increased capillary density, observed in both PlGF and PlGF/RGS4 mice prior to TAC was sustained for 6 weeks after TAC in both models (Fig. 5e). Thus, these results indicate that PlGF-induced angiogenesis protects against early but not late contractile dysfunction after LV pressure overload.

### miR-182 is not associated with LV pressure overload or pathological hypertrophy

**Figure 6:**
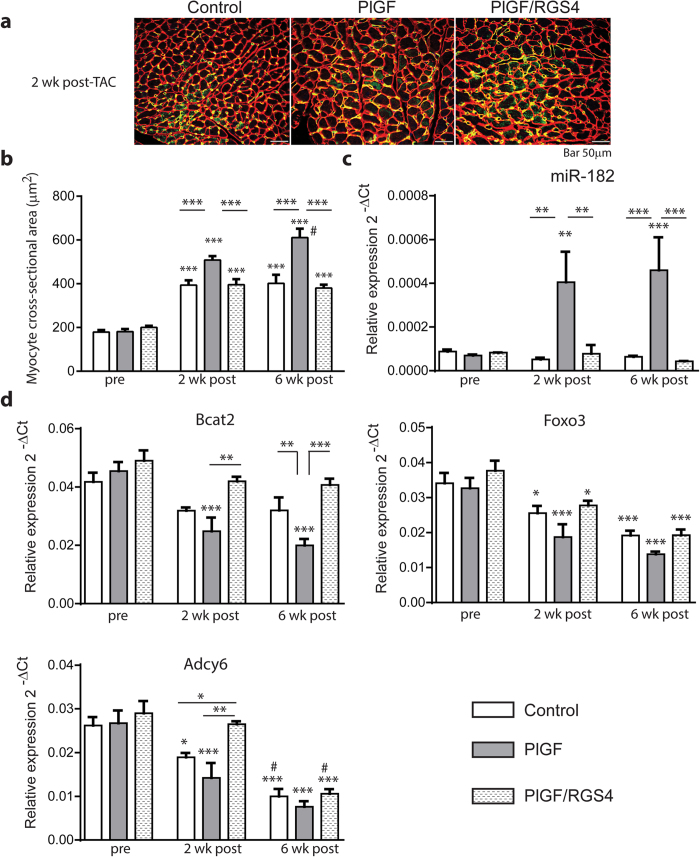
miR-182 is not associated with the hypertrophic response induced by LV pressure overload. (**a**) Representative immunostaining with anti CD31 and anti-laminin Abs of LV myocardium sections at 2 weeks post-TAC in PlGF, PlGF/RGS4 and control mice. (**b**) Increased cardiomyocyte cross-sectional area in LV myocardium sections in all groups after TAC. Greater cardiomyocyte area in PLGF mice. (**c**) Increased miR-182 expression in PlGF mice, but not in PlGF/RGS4 or control mice. (**d**) Comparative analysis of Bcat2, Foxo3 and Adcy6 expression prior and during TAC. n = 6 (control pre-TAC); 6 (PlGF pre-TAC); 6 (PlGF/RGS4 pre-TAC); n = 7 (control 2 wk post-TAC); 4 (PlGF 2 wk post-TAC); 4 (PlGF/RGS4 2 wk post-TAC); 6 (control 6 wk post-TAC); 6 (PlGF 6 wk post-TAC); 5 (PlGF/RGS4 6 wk post-TAC). *P < 0.05, **P < 0.01, ***P < 0.001 vs. pre-TAC or as indicated; # *P *< 0.05 vs. 2 wk post-TAC.

Next, we assessed the expression of miR-182 and targeted genes in pathological hypertrophy induced by LV pressure overload. The greater hypertrophic response in PlGF mice after TAC was also associated with greater cardiomyocyte area when compared with control and PlGF/RGS4 mice at both 2 and 6 weeks post-TAC (Fig. 6a,b). Consistent with our observations that miR-182 modulates the angiogenesis-induced hypertrophic response, there was also a 7-fold increase in miR-182 expression in PlGF mice at both time points (Fig. 6c). In contrast, despite the induction of hypertrophy after TAC, there was no increase in miR-182 expression in either control or PlGF/RGS4 mice (Fig. 6c). The lack of miR-182 induction after TAC is in accord with *in vitro* data showing that AngII, a cardiac hypertrophic GPCR agonist, also did not induce miR-182 expression (Fig. 2a). Thus, miR-182 upregulation does not appear to be associated with pressure overload-induced or pathological hypertrophy.

We also analyzed miR-182 targeted genes in PlGF, PlGF/RGS4 and control mice at 2 and 6 weeks post-TAC. We found a significant and persistent downregulation of Bcat2, Foxo3 and Adcy6 transcripts in PlGF mice at 2 and 6 weeks after TAC, compared to pre-TAC measurements. (Fig. 6d). Despite the lack of increase in miR-182, pressure overload significantly decreased the expression of Adcy6 and Foxo3 in control and PlGF/RGS4 mice (Fig. 6d). In the case of Adcy6, the expression in PlGF mice at 2 weeks post-TAC was comparable with that in controls, although it was prevented in PlGF/RGS4 mice. In contrast, at 6 weeks after TAC, Adcy6 expression was equally decreased in all groups. Whereas Foxo3 downregulation after TAC was similar in all groups at both time points. These findings show that Adcy6 and Foxo3 are downregulated in response to pressure overload with a greater reduction in PlGF mice, suggesting an additive effect of pressure overload and miR-182. In contrast, Bcat2 expression was strikingly lower at 2 and 6 weeks post-TAC in PlGF mice, but there was only a trend to decreased expression in control and PlGF/RGS4 mice (Fig. 6d). These results indicate that the downregulation of Bcat2 strongly relates to the increase in miR-182 expression that is present in PlGF, but not in PlGF/RGS4 or control mice.

### Bcat2 deficiency promotes hypertrophy and Akt/mTORC1 activation in cardiomyocytes

**Figure 7:**
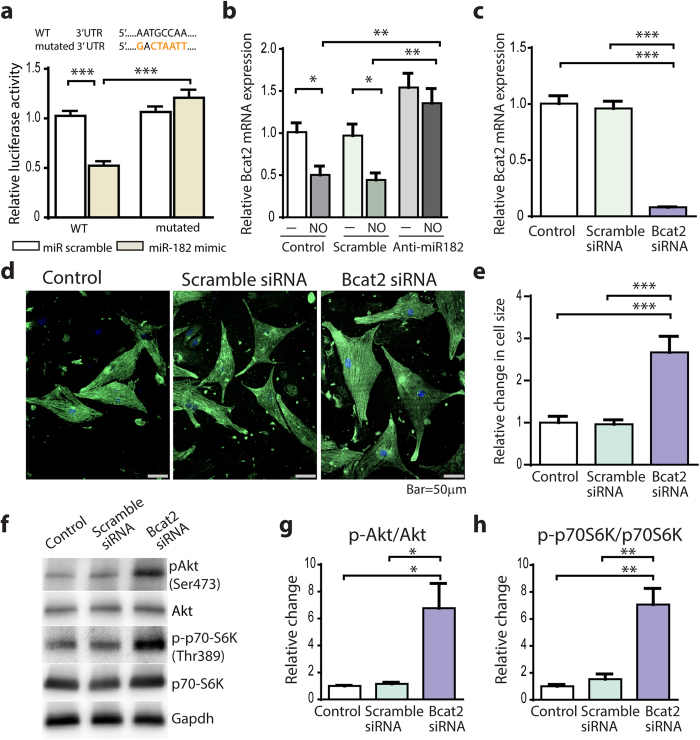
Bcat2 depletion induces cardiomyocyte hypertrophy. (**a**) Reduced activity of wild type (WT) Bcat2-3′UTR luciferase reporter in HEK293T cells co-transfected with miR-182 mimic, compared with miR-scramble. miR-182 did not reduce the activity of mutant (Mut) Bcat2-3′UTR luciferase reporter. Mutations in Bcat2-3′UTR are indicated in orange. (**b**) Reduction of Bcat2 expression in NO-treated NRCs and restoration of Bcat2 expression with anti-miR-182 treatment. (**c**) Bcat2 depletion in Bcat2-siRNA-treated NRCs. (**d**) Representative Bcat2-siRNA-treated NRCs after 24 hrs in a serum-free medium, compared with scramble-siRNA treated and control NRCs (co-staining with anti-α-actinin sarcomeric Ab and DAPI). (**e**) Increased cell surface area of Bcat2-siRNA-treated NRCs. (**f**) Western blot analysis of Akt^Ser473^ and p70-S6K^Thr389^. (**g**) Relative change in Akt activation at Akt^Ser473^. (**h**) Relative change in p70-S6K activation at p70-S6K^Thr389^. n = 3 independent experiments; * *P* < 0.05; **P < 0.01; ***P < 0.001.

Because published studies have already shown that miR-182 directly regulates the expression of Foxo3^16^,^17^,^18^ and Adcy6^19^,^20^, we further investigated whether miR-182 directly regulates the expression of Bcat2. To determine the direct binding of miR-182 to Bcat2-3′ UTR, we co-transfected HEK293T cells with wild-type or mutant Bcat2-3′ UTR reporter plasmid and either a miR-182 mimic or a miR-scramble negative control. The miR-182 mimic reduced the luciferase activity of wild type but not mutant Bcat2-3′ UTR reporter, compared with cells transfected with control miR-scramble (Fig. 7a). Next, we hypothesized that miR-182 negatively regulates Bcat2 expression in NO-treated NRCs and that the treatment with anti-miR-182 restores Bcat2 expression and inhibits hypertrophic response. Indeed, in NO-treated NRCs, the upregulation of miR-182 (Fig. 2b) suppressed Bcat2 expression by 50% (Fig. 7b). Consequently, the treatment of NO-treated NRCs with anti-miR-182 restored Bcat2 expression (Fig. 7b) and prevented the hypertrophic response (Fig. 2c,d). These data are in agreement with *in vivo* observations of reduced Bcat2 expression in PlGF mice concurrently with the upregulation of miR-182 and the development of hypertrophy and complete restoration of Bcat2 expression and inhibition of hypertrophic response with anti-miR-182 treatment (Figs 3 and 4b). Next, in order to determine whether Bcat2 has a direct effect on the cell growth and the Akt/mTORC1 pathway, we silenced Bcat2 expression in NRCs with siRNA (Fig. 7c). Interestingly, Bcat2 depletion resulted in a 2.5 fold increase in cardiomyocyte size (Fig. 7d,e) and a 7 fold increase in phosphorylation of Akt^Ser473^ and p70-S6K^Thr389^ (Fig. 7 f–h). Because Bcat2 is a critical determinant of the cellular catabolism of BCAAs our data strongly suggest that a loss in Bcat2 stimulates the Akt and mTORC1/p70-S6K activation and the hypertrophic response through a BCAA-dependent mechanism.

## Discussion

These data are the first to show that endothelium to cardiomyocyte communication can induce the expression of miRNAs in the heart, and that miR-182 induction is critical for the hypertrophic response that links angiogenesis to cardiomyocyte growth. We found that miR-182 upregulation in the heart occurs subsequent to PlGF-induced angiogenesis and requires endothelial-derived NO production, which triggers RGS4 degradation in cardiomyocytes. Consequently, the expression of miR-182 and myocardial hypertrophy were inhibited by the concomitant transgenic expression of PlGF and RGS4, or the expression of PlGF in eNOS^−/−^ mice. The mechanism by which miR-182 expression is controlled downstream of RGS4 is not known and future studies are required to address this issue.

For the purpose of this study, we focused on miR-182 and its important downstream role to modulate the Akt/mTORC1 pathway. Our data show that miR-182 inhibition with antagomir prevented phosphorylation at Akt^Ser473^ and p70-S6K^Thr389^ and hypertrophy in both NO-treated cardiomyocytes and PlGF mice, despite the loss in RGS4 protein. The present data, also provide insight into potential mechanisms by which miR-182 might regulate the hypertrophic response, including suppression of Bcat2, Foxo3 and Adcy6 expression. Remarkably, the depletion of Bcat2, an enzyme involved in the mitochondrial catabolism of BCAAs, had a direct effect on cardiomyocyte hypertrophy and increased Akt^Ser473^ and p70-S6K^Thr389^ phosphorylation, indicating the potential contribution of BCAAs to the growth response.

Angiogenic factor PlGF binds to VEGFR1 and indirectly amplifies angiogenesis by displacing VEGF-A from VEGFR1 and making it available for binding to pro-angiogenic VEGFR2^21^. As we^11^ and others^10^ have observed, PlGF did not directly promote cardiomyocyte hypertrophy, which might be explained by the lack of VEGFR1 expression on cardiomyocytes^8^,^10^. Consistent with the lack of a direct hypertrophic effect, PlGF also did not induce miR-182 expression in cardiomyocytes. Another member of the VEGF family, VEGFB, binds to VEGFR1, but is less pro-angiogenic, also promotes myocardial hypertrophy in the rat heart^12^. However, in contrast with our PlGF model, VEGFB-induced hypertrophy was independent of NO^12^. This apparent mechanistic difference between PlGF and VEGFB may reflect the capabilities of PlGF to induce angiogenesis and to activate endothelial eNOS through VEGFR1 phosphorylation at Tyr794^22^.

Only a few previous studies have addressed the potential function of miR-182 in the heart. One report related miR-182 to cardiac hypertrophy in rejecting cardiac allografts^23^, while other two provided evidences of increased circulated miR-182 in patients undergoing coronary artery bypass surgery^24^ and patients with chronic congestive heart failure^25^. However, several studies showed that miR-182 functions as an oncomiR in the early stages to promote cancer cell proliferation and growth and as a tumor suppressor in the late stages to inhibit cancer metastasis^18^ Among multiple targets, miR-182 oncogenicity was associated with the repression of FOXO3^17^,^18^ and FOXO1^26^.

Our findings reveal that miR-182 suppressed Bcat2, Foxo3 and Adcy6 expression, raising the possibility that one or more of these actions contributed to the angiogenesis-induced hypertrophic response in the heart. Downregulation of Foxo3, a regulator of the atrophy-related gene program^27^ would be expected to promote a growth response. Pressure overload and hypertrophic GPCR agonists are known to downregulate Foxo3 through the action of miR-23a^28^. However, a recent study provides evidence that miR-182 also directly decreases Foxo3 expression. During glucocorticoid-induced atrophy in skeletal muscle, a decrease of intracellular miR-182 and increased release of miR-182 into exosomes augmented the Foxo3 expression and activation of the atrophy-related gene expression^16^. The overexpression of miR-182 decreased Foxo3 expression and attenuated skeletal muscle atrophy^16^. Together with our findings, these data indicate that miR-182 favors cellular growth in skeletal and cardiac muscle.

Bcat2 is another potential mechanism that might contribute to the hypertrophic response induced by miR-182. Bcat2 catalyzes the first step in mitochondrial catabolism of BCAAs (leucine, isoleucine, valine) and therefore is a critical determinant of the cellular BCAA content. BCAAs are avidly extracted by the heart in patients with coronary artery disease^29^ and oxidized after conversion to their ketoacids. Interestingly, a recent study highlighted the role of Bcat2 in glucocorticoid-induced atrophy in skeletal muscle. In this model, upregulation of Bcat2 increased BCAA catabolism and induced muscle atrophy, while conversely BCAA treatment activated mTORC1 and attenuated muscle atrophy^30^. Studies done in Bcat2^−/−^ mice showed also that elevated BCAAs and/or loss of BCAA catabolism increased the protein synthesis and mTORC1/p70S6K in skeletal muscle^31^. The anabolic effect of amino acids, in particular BCAAs to increase protein synthesis is mediated by their direct activation of mTORC1 with subsequent downstream phosphorylation of p70-S6K at Thr389^32^,^33^. BCAAs appear to promote Akt phosphorylation at Ser473 through the activation of mTORC2 in a manner dependent on the activity of class I PI3K^34^. Our results expand upon these findings by showing that miR-182 directly regulates Bcat2 expression and that Bcat2 downregulation activates Akt^Ser473^ and mTORC1/p70-S6K^Thr389^ and promotes cardiomyocyte hypertrophy.

Previously, it has been shown that miR-182 directly modulates Adcy6 expression to regulate mammalian circadian rhythm^19^,^20^. However, the contribution of Adcy6 (required for cAMP synthesis in response to β-adrenergic stimulation) to the cardiomyocyte hypertrophy is not well understood. Although, Adcy6 expression was decreased in myocardial infarction and chronic LV pressure overload, this event was linked to a severe impairment in both calcium signaling and LV contractile function^35^,^36^.

We did not find evidence of miR-182 induction during either LV pressure overload *in vivo* or in response to the GPCR agonist Ang II *in vitro*. Interestingly, pressure overload did downregulate Foxo3 and Adcy6 presumably through a distinct mechanism(s). However, in the case of Bcat2 a greater reduction was associated with miR-182 than that detected with pressure overload. Thus, angiogenesis-induced and pressure overload-induced hypertrophy may have some convergence and thus additive effects on selected downstream miR-182 targets.

Interestingly, during TAC-induced pressure overload, superimposed effect of angiogenesis on hypertrophy resulted in a greater myocardial hypertrophic response. Initially, this was protective at 2 weeks after TAC, preserving contractile function, but later at 6 weeks appeared to be detrimental. Although angiogenesis is more robust during TAC in PlGF mice, it still might not be sufficient to compensate for the excessive hypertrophy that develops in this model. Alternatively, other pathological factors and/or the duration of stress stimuli may contribute to the decline in heart function. The latter hypothesis is supported by a previous observation that pathologic exposure to intermittent pressure overload can trigger cardiac dysfunction independent of hypertrophy *per se*^37^. Furthermore, increased angiogenesis during pressure overload-induced hypertrophy did not prevent the contractile dysfunction in a mouse model with sustained expression of peroxisome proliferator-activated receptor γ coactivator-1α (PGC-1α)^38^. PGC-1α was shown to regulate the VEGF-induced angiogenesis in response to exercise and ischemia^39^.

Cardiomyocyte transgenic PlGF expression has been previously reported to exaggerate the myocardial hypertrophic response after TAC^10^. However, in contrast to our model, there was no baseline induction of angiogenesis or hypertrophy in the prior experiments^10^. This apparent discrepancy likely reflects the different promoters used to control the transgene expression: traditional CMV minimal promoter coupled with TRE in our model^11^ versus a modified MHC promoter linked to TRE to induce a low level of transgene expression in the prior study^10^. Interestingly, in contrast with our data, the PlGF transgenic mice, in the prior study, did not develop greater LV contractile dysfunction during TAC, even with superimposed angiotensin II/phenylephrine infusion^10^. Genetic background differences in the mouse strains could also contribute to these results: C57BL/6 strain in our study versus FVB/N strain, more resistant to cardiac dysfunction in the prior study^10^.

Although our study has implicated cardiomyocyte miR-182 as a target of endothelium to cardiomyocyte communication, we cannot exclude the possibility that miR-182 may conversely modulate cardiomyocyte to endothelial cell communication and promote angiogenesis. A recent study showed that the conditioned medium from miR-182 transfected liver cancer cell lines promoted HUVEC angiogenesis potentially related to downregulation of RAS p21 protein activator 1 (RASA1)^40^. To the extent that miR-182 promotes an increase in vasculature and blood perfusion, it could provide an additional mechanism responsible for developing and sustaining myocardial hypertrophy. However, more work is warranted to elucidate this potential crosstalk mechanism.

In conclusion, miR-182 is a novel target of endothelium to cardiomyocyte communication and plays an important role in the activation of the Akt/mTORC1 pathway and the hypertrophic response induced by angiogenesis in heart.

## Methods

### Animal models

The TET-OFF mouse lines PlGF and PlGF/RGS4 with regulated heart specific transgenic expression sensitive to tetracycline transactivator (tTA) driven by the αMHC promoter were recently described by our laboratory^11^. All transgenic mouse lines were generated in C57Bl/6 strain. The PlGF and PlGF/RGS4 were generated by crossing driver line αMHC-tTA^7^ with TRE-PlGF and correspondingly TRE-PlGF/RGS4 mice. The PlGF-induced eNOS^−/−^ mice were generated by crossing αMHC-tTA- eNOS^−/−^with TRE-PlGF- eNOS^−/−^ mice. Genotyping was performed by Transnetyx (Cordova), using a qPCR based system to detect the presence or absence of a target sequence within each sample^11^. Transgene expression was repressed during embryonic and postnatal development by doxycycline in diet (200 mg/kg; Bio-Serv) and induced on normal chow diet at 5 weeks of age. Littermates inheriting only responder or driver transgene were used as controls. All animal experiments were performed under a protocol approved by the Institutional Animal Care and Use Committee of Yale University. All experiments were carried out in accordance with the approved guidelines.

### Transverse aortic constriction (TAC)

PlGF, PlGF/RGS4 and control mice, 8 weeks old, were subjected to TAC by tying an 8-0 nylon suture ligature against a 27.5 gauge blunt needle, as previously described^41^. The resulting constriction produces a 4 m/sec velocity. Mice were monitored up to 6 weeks after TAC procedure.

### Echocardiography

Mice were anesthetized with 1% isoflurane inhaled anesthesia. M-mode images were acquired with a Visual Sonic 2100 high-resolution ultrasound imaging system^11^.

### Antagomir treatment in mice.

The *in vivo* LNA anti-mmu-miR-182-5p probe and control miR-scramble (Exiqon) were packed into the MaxSuppressor^TM^. *In Vivo* RNA-LANCEr II, a phospholipid-oil emulsion (Bioo Scientific). Prior to *in vivo* administration in order to increase efficiency of delivery, the lipid suspension was homogenized to unilamellar vesicles, 120–140 nm diameter range, by extrusion through a 100 nm pore size filter using a Lipid Extruder (Bioo Scientific). LNA anti-miR-182 and control miR-scramble were delivered via tail vein injection, 20 μg/mouse/ injection on day 1, 4 and 7 during 3 weeks treatment period.

### miRNA isolation and quantification

Total RNA was isolated from LV samples using miRNeasy mini kit (Qiagen) and converted into cDNA using a TaqMan MicroRNA Reverse Transcription kit (Applied Biosystems) with specific miRNA primers supplied with the TaqMan MicroRNA Assay (Applied Biosystems). Quantitative PCR was performed in a CFX96 Real-time PCR detection system (Bio-Rad) using Taqman miRNA probes (Applied Biosystems) and SsoFast Probes Supermix (Bio-Rad). Small nucleolar RNA 202 or U6 (Applied Biosystems) were used as endogenous control for miRNA quantification.

### miRNA and mRNA microarrays

We used the Affymetrix GeneChip miRNA 2.0 array for miRNA expression profiling. The arrays were processed in conjunction with the FlashTag Biotin Labelling kit (Genisphere) by W.M. Keck Biotechnology Resource Laboratory at Yale. Total RNA (130 ng) isolated from LV samples were labeled with the FlashTag Biotin HSR RNA Kit, according to the manufacturer’s recommendation. Biotin-labeled samples were hybridized for 16 hours at 48 °C and 60 rpm on GeneChip miRNA 2.0 arrays. GeneChips were washed and stained in an Affymetrix GeneChip Fluidics Station 450, then scanned with a GeneChip Scanner 3000 7G. The data were analyzed and normalized using Expression Console Software with Affymetrix analysis settings.

We used the Affymetrix mouse gene 1.0 ST arrays (~29000 transcripts) for mRNA expression profiling. The arrays were processed and analyzed by W.M. Keck Biotechnology Resource Laboratory at Yale. High quality total RNA (250 ng) samples were processed to biotin-labeled cDNA using an Ambion WT expression kit and Affymetrix GeneChip WT Terminal Labeling kit according to the manufacturer’s instructions. Biotin-labeled samples were hybridized for 16 hours at 45 °C and 60 rpm on GeneChip Mouse Gene 1.0 ST Array. GeneChips were washed and stained in an Affymetrix Fluidics Station 450, then scanned using a GeneChip Scanner 3000 7G. The data were analyzed with a Microarray Suite version 5.0 (MAS 5.0) using Affymetrix analysis settings and global scaling as normalization method.

The data have been deposited in NCBI’s Gene Expression Omnibus^42^ and are accessible through GEO Series accession number GSE67824.

### miR-182 detection and localization by *in situ* hybridization (ISH)

ISH was performed on LV sections, fixed in 4% PFA and embedded in paraffin, using a specific double-digoxigenin (DIG) labeled miRCURY LNA mmu-miR-182 detection probe (Exiqon). DIG labeled miR-scramble probe was used as a negative control and a DIG labeled probe against U6 snRNA as a positive control. The ISH detection was optimized for 50 nM miR-182 and miR-control probe and 25 nM for U6 positive control. After ISH the sections were co-stained with an anti-cardiac troponin T antibody (Abcam) and DAPI. The images were analyzed with a Nikon Eclipse 80i microscope.

### Histological assessments

*Capillary density* and *cardiomyocyte size* were determined on serial 5 μm LV sections immunostained with anti CD31 antibody (R&D Systems) or IB4 (Sigma) and anti laminin Ab (Sigma), as we previously described^7^,^11^. Capillary/myocyte ratio and cardiomyocyte cross-sectional area were determined in captured images using a Nikon Eclipse 80i microscope and analyzed with the Image J analysis software.

### Cell culture experiments

*Neonatal rat cardiomyocytes* (NRCs) were isolated from 2-day-old Sprague-Dawley rats using a Neonatal Cardiomyocyte Isolation System (Worthington Biochemical Corp.) and plated on fibronectin-coated plates in DMEM supplemented with 10% FBS^7^,^11^. NRCs were treated with NO donor DETA-NONOate (Cayman Chemical Co), PlGF-2 (R&D System) or Ang II (Sigma-Aldrich) in serum-free medium (DMEM supplemented with 1 mg/ml BSA, 10 μg/ml transferrin and 10 μg/ml insulin) for 24 hours, as we previously described^11^.

*Transfection of NRCs* with anti-rno-miR-182 5p (Ambion), rno-miR-182 mimic 5p (Ambion), control miR scramble (Ambion), Bcat2 siRNA (Ambion), and AllStars Negative Control siRNA (Qiagen) were carried out using Lipofectamine RNAiMAX (Invitrogen). After transfection, NRCs were kept overnight in complete medium, then were placed in serum-free medium and used for experiment.

*NRCs surface area*. For cell area measurements, NRCs were plated on fibronectin-coated glass bottom plates and fixed at the end of experiments with 4% ice-cold PFA. After permeabilization with 2% PFA, 0.1% Triton X-100 and 0.1% NP-40, the cells were stained with an anti-α-actinin sarcomeric Ab (Sigma-Aldrich) and DAPI (Invitrogen) as previously described^11^. NRCs were visualized with a Nikon Ti E Eclipse inverted spinning disk confocal microscope. Cell surface area was determined in 12 random fields (20 × 1.5 magnification) captured with a Volocity 3D imaging software (PerkinElmer) and then measured using an Image J analysis software.

*3′-UTR luciferase reporter assay.* To determine whether miR-182 directly binds to the Bcat2-3’ UTR, we utilized a Gaussia luciferase (GLuc) /secreted Alkaline Phosphatase (SEAP) dual-reporter pEZX-MT05 vector (GeneCopoeia). HEK293T cells were co-transfected with 1 μg of wild type or mutated Bcat2-3′UTR GLuc/SEAP dual-reporter pEZX-MT05 plasmid and mmu-miR-182-5p mimic (Ambion) or control miR-scramble (Ambion) (100 nM) with Lipofectamine 2000 (Invitrogen). After 48 hours, both the GLuc and internal control SEAP activity were assessed in cell culture medium using a GeneCopoeia Secrete-Pair Dual Luminescence assay kit and measured with a BioTek Synergy 2 Multi-Mode microplate reader. Luciferase activity was normalized to SEAP activity and reported relative to control miR-scramble activity.

*Transfection of MEFs*with mmu-miR-182-5p mimic (Ambion) (50 nM) and control miR scramble (Ambion) (50 nM) were carried out using Lipofectamine RNAiMAX (Invitrogen). After transfection, MEFs were kept overnight in complete medium (DMEM supplemented with 10% FBS), then were placed in serum-free medium and analyzed 48 hours later.

### Endothelial cell sprouting assay

HUVECs were transfected with hsa-miR-182 5p mimic or miR scramble and the sprouting assay was conducted as previously described^43^. Briefly, 24 hours after transfection the cells were harvested and re-suspended in 300 μl fibrinogen solution (2.5 mg/ml fibrinogen (Sigma) in EBM-2 supplemented with 2% FBS and 50 μg/ml aprotinin (Sigma)), and plated on top of a pre-coated fibrin layer (400 μl fibrinogen solution clotted with 1U thrombin (Sigma) for 20 min at 37C). The second layer of fibrin was added and allowed to clot for 1 hour at 37 °C. Human fibroblasts, WI-38 cells (250,000 cells/well), in EBM-2 supplemented with 2% FBS and 25 ng/ml VEGF-A, were then plated on top of the second fibrin layer. Cultures were then incubated at 37 °C, 5% CO_2_. After 5 days, the cultures were labeled with 4 μg/ml Calcein AM for 1 hour, and imaged with a Nikon Ti E Eclipse inverted spinning disk confocal microscope using a standard FITC filter.

### miRNA target genes expression

Total RNA was reversed transcribed using iScript cDNA synthesis kit (Bio-Rad) and qPCR was carried out using iQ SYBR Green supermix kit (Bio-Rad). The following primer sets were used:

**Foxo3 mouse (**NM_019740.2): F: 5′cgttgttggtttgaatgtgg 3′and R: 5′ gagagcagatttggcaaagg 3′; **Adcy6 mouse**: (NM_007405): F: 5′agggaggtcctgtgtgtttg 3′and R: 5′ctcctgtgcaacctgggtat 3′; **Bcat2 mouse (**NM_009737): F: 5′gcatctagtccagcgtcctc 3′and R: 5′ccaaggttctcccttgaaca 3′, **Bcat2 rat** (NM_022400.1): F: 5′caggatgctacgttctgcaa 3′ and R: 5′aacaggagggcttgtgtgac 3′; **Gapdh mouse** (NM_008084.2): F: 5′ aactttggcattgtggaagg 3′and R: 5′ acacattgggggtaggaaca 3′. **Gapdh rat** (NM_017008): F: 5′ agacagccgcatcttcttgt 3′ and R: 5′ cttgccgtgggtagagtcat 3′.

### Western blotting

LV tissue/cell homogenates were extracted in RIPA lysis buffer as previously described^7^,^11^. Following transfer to Immobilon PVDF membranes (Perkin Elmer), the membranes were immunoblotted with specific antibodies against: RGS4 (Millipore), Akt (Millipore); Akt^Ser473^, p70-S6K and p70-S6K^Thr389^ (Cell Signaling); GAPDH (Research Diagnostics, INC). Immunoreactive bands were visualized using the horseradish peroxidase-conjugated secondary antibody and enhanced chemiluminescent substrate (Pierce). Images were captured using a G: Box gel imaging system and analyzed with a GeneTools software (Syngene).

### Statistical analysis

Data are presented as mean ± SEM. Differences between multiple groups were assessed, as required by the experimental design, using one-way ANOVA, two-way ANOVA or repeat measures two-way ANOVA followed by Tukey’s post-hoc test for multiple comparisons. Comparisons between two independent groups were performed using a two-sample t test. All *p* values were calculated using two-tailed statistical tests. Differences were considered significant when *p *< 0.05. Data were analyzed using GraphPad Prism Version 6.03.

## Additional Information

**How to cite this article**: Li, N. *et al.* miR-182 Modulates Myocardial Hypertrophic Response Induced by Angiogenesis in Heart. *Sci. Rep.* **6**, 21228; doi: 10.1038/srep21228 (2016).

## Supplementary Material

Supplementary Information

## References

[b1] Tirziu D., Giordano F. J. & Simons M. Cell communications in the heart. Circulation 122, 928–937 (2010). 20805439 10.1161/CIRCULATIONAHA.108.847731 PMC2941440

[b2] Izumiya Y. *et al.* Vascular endothelial growth factor blockade promotes the transition from compensatory cardiac hypertrophy to failure in response to pressure overload. Hypertension 47, 887–893 (2006). 16567591 10.1161/01.HYP.0000215207.54689.31 PMC3132898

[b3] Shiojima I. *et al.* Disruption of coordinated cardiac hypertrophy and angiogenesis contributes to the transition to heart failure. J Clin Invest 115, 2108–2118 (2005). 16075055 10.1172/JCI24682 PMC1180541

[b4] Walsh K. & Shiojima I. Cardiac growth and angiogenesis coordinated by intertissue interactions. J Clin Invest 117, 3176–3179 (2007). 17975662 10.1172/JCI34126 PMC2045631

[b5] Heineke J. *et al.* Cardiomyocyte GATA4 functions as a stress-responsive regulator of angiogenesis in the murine heart. J Clin Invest 117, 3198–3210 (2007). 17975667 10.1172/JCI32573 PMC2045611

[b6] Tomanek R. J., Doty M. K. & Sandra A. Early coronary angiogenesis in response to thyroxine: growth characteristics and upregulation of basic fibroblast growth factor. Circ Res 82, 587–593 (1998). 9529163 10.1161/01.res.82.5.587

[b7] Tirziu D. *et al.* Myocardial hypertrophy in the absence of external stimuli is induced by angiogenesis in mice. J Clin Invest 117, 3188–3197 (2007). 17975666 10.1172/JCI32024 PMC2045601

[b8] Roncal C. *et al.* Beneficial effects of prolonged systemic administration of PlGF on late outcome of post-ischaemic myocardial performance. J Pathol 216, 236–244 (2008). 18729077 10.1002/path.2408

[b9] Bry M. *et al.* Vascular endothelial growth factor-B acts as a coronary growth factor in transgenic rats without inducing angiogenesis, vascular leak, or inflammation. Circulation 122, 1725–1733 (2010). 20937974 10.1161/CIRCULATIONAHA.110.957332

[b10] Accornero F. *et al.* Placental growth factor regulates cardiac adaptation and hypertrophy through a paracrine mechanism. Circ Res 109, 272–280 (2011). 21636802 10.1161/CIRCRESAHA.111.240820 PMC3146170

[b11] Jaba I. M. *et al.* NO triggers RGS4 degradation to coordinate angiogenesis and cardiomyocyte growth. J Clin Invest 123, 1718–1731 (2013). 23454748 10.1172/JCI65112 PMC3613910

[b12] Kivela R. *et al.* VEGF-B-induced vascular growth leads to metabolic reprogramming and ischemia resistance in the heart. EMBO Mol Med 6, 307–321 (2014). 24448490 10.1002/emmm.201303147 PMC3958306

[b13] Hu R. G. *et al.* The N-end rule pathway as a nitric oxide sensor controlling the levels of multiple regulators. Nature 437, 981–986 (2005). 16222293 10.1038/nature04027

[b14] Mittmann C. *et al.* Expression of ten RGS proteins in human myocardium: functional characterization of an upregulation of RGS4 in heart failure. Cardiovasc Res 55, 778–786 (2002). 12176127 10.1016/s0008-6363(02)00459-5

[b15] Dweep H., Sticht C., Pandey P. & Gretz N. miRWalk–database: prediction of possible miRNA binding sites by “walking” the genes of three genomes. J Biomed Inform 44, 839–847 (2011). 21605702 10.1016/j.jbi.2011.05.002

[b16] Hudson M. B. *et al.* miR-182 attenuates atrophy-related gene expression by targeting FoxO3 in skeletal muscle. Am J Physiol Cell Physiol 307, C314–C319 (2014). 24871856 10.1152/ajpcell.00395.2013 PMC4137139

[b17] Segura M. F. *et al.* Aberrant miR-182 expression promotes melanoma metastasis by repressing FOXO3 and microphthalmia-associated transcription factor. Proc Natl Acad Sci USA 106, 1814–1819 (2009). 19188590 10.1073/pnas.0808263106 PMC2634798

[b18] Yang W.-B. *et al.* Sp1-mediated microRNA-182 expression regulates lung cancer progression. Oncotarget 5, 740–753, (2014). 24519909 10.18632/oncotarget.1608 PMC3996653

[b19] Saus E. *et al.* Genetic variants and abnormal processing of pre-miR-182, a circadian clock modulator, in major depression patients with late insomnia. Hum Mol Genet 19, 4017–4025 (2010). 20656788 10.1093/hmg/ddq316

[b20] Xu S., Witmer P. D., Lumayag S., Kovacs B. & Valle D. MicroRNA (miRNA) transcriptome of mouse retina and identification of a sensory organ-specific miRNA cluster. J Biol Chem 282, 25053–25066 (2007). 17597072 10.1074/jbc.M700501200

[b21] Autiero M. *et al.* Role of PlGF in the intra- and intermolecular cross talk between the VEGF receptors Flt1 and Flk1. Nat Med 9, 936–943 (2003). 12796773 10.1038/nm884

[b22] Ahmad S. *et al.* Direct evidence for endothelial vascular endothelial growth factor receptor-1 function in nitric oxide-mediated angiogenesis. Circ Res 99, 715–722 (2006). 16946136 10.1161/01.RES.0000243989.46006.b9

[b23] Wei L. *et al.* Differential expression of microRNAs during allograft rejection. Am J Transplant 12, 1113–1123 (2012). 22300508 10.1111/j.1600-6143.2011.03958.x PMC3461331

[b24] Taurino C. *et al.* Gene expression profiling in whole blood of patients with coronary artery disease. Clin Sci (Lond) 119, 335–343 (2010). 20528768 10.1042/CS20100043 PMC2922838

[b25] Cakmak H. A. *et al.* The prognostic value of circulating microRNAs in heart failure: preliminary results from a genome-wide expression study. J Cardiovasc Med (Hagerstown, Md) 16, 431–437 (2015). 10.2459/JCM.0000000000000233 25643195

[b26] Guttilla I. K. & White B. A. Coordinate regulation of FOXO1 by miR-27a, miR-96, and miR-182 in breast cancer cells. J Biol Chem 284, 23204–23216 (2009). 19574223 10.1074/jbc.M109.031427 PMC2749094

[b27] Skurk C. *et al.* The FOXO3a transcription factor regulates cardiac myocyte size downstream of AKT signaling. J Biol Chem 280, 20814–20823 (2005). 15781459 10.1074/jbc.M500528200 PMC3632436

[b28] Wang K. *et al.* Cardiac Hypertrophy Is Positively Regulated by MicroRNA miR-23a. J Biol Chem 287, 589–599 (2012). 22084234 10.1074/jbc.M111.266940 PMC3249113

[b29] Young L. H. *et al.* Myocardial protein turnover in patients with coronary artery disease. Effect of branched chain amino acid infusion. J Clin Invest 87, 554–560 (1991). 1991838 10.1172/JCI115030 PMC296343

[b30] Shimizu N. *et al.* Crosstalk between Glucocorticoid Receptor and Nutritional Sensor mTOR in Skeletal Muscle. Cell Metabolism 13, 170–182 (2011). 21284984 10.1016/j.cmet.2011.01.001

[b31] She P. *et al.* Disruption of BCATm in Mice Leads to Increased Energy Expenditure Associated with the Activation of a Futile Protein Turnover Cycle. Cell Metabolism 6, 181–194 (2007). 17767905 10.1016/j.cmet.2007.08.003 PMC2693888

[b32] Blomstrand E., Eliasson J., Karlsson H. K. R. & Köhnke R. Branched-Chain Amino Acids Activate Key Enzymes in Protein Synthesis after Physical Exercise. J Nutri 136, 269S–273S (2006). 10.1093/jn/136.1.269S 16365096

[b33] Sancak Y. *et al.* Ragulator-Rag complex targets mTORC1 to the lysosomal surface and is necessary for its activation by amino acids. Cell 141, 290–303 (2010). 20381137 10.1016/j.cell.2010.02.024 PMC3024592

[b34] Tato I., Bartrons R., Ventura F. & Rosa J. L. Amino acids activate mammalian target of rapamycin complex 2 (mTORC2) via PI3K/Akt signaling. J Biol Chem 286, 6128–6142 (2011). 21131356 10.1074/jbc.M110.166991 PMC3057817

[b35] Espinasse I. *et al.* Decreased type VI adenylyl cyclase mRNA concentration and Mg(2+)-dependent adenylyl cyclase activities and unchanged type V adenylyl cyclase mRNA concentration and Mn(2+)-dependent adenylyl cyclase activities in the left ventricle of rats with myocardial infarction and longstanding heart failure. Cardiovasc Res 42, 87–98 (1999). 10434999 10.1016/s0008-6363(98)00283-1

[b36] Sugano Y. *et al.* Activated expression of cardiac adenylyl cyclase 6 reduces dilation and dysfunction of the pressure-overloaded heart. Biochem Biophys Res Commun 405, 349–355 (2011). 21195051 10.1016/j.bbrc.2010.12.113 PMC3219303

[b37] Perrino C. *et al.* Intermittent pressure overload triggers hypertrophy-independent cardiac dysfunction and vascular rarefaction. J Clin Invest 116, 1547–1560 (2006). 16741575 10.1172/JCI25397 PMC1464895

[b38] Pereira R. O. *et al.* Maintaining PGC-1alpha expression following pressure overload-induced cardiac hypertrophy preserves angiogenesis but not contractile or mitochondrial function. FASEB J 28, 3691–3702 (2014). 24776744 10.1096/fj.14-253823 PMC4101649

[b39] Arany Z. *et al.* HIF-independent regulation of VEGF and angiogenesis by the transcriptional coactivator PGC-1α. Nature 451, 1008–1012 (2008). 18288196 10.1038/nature06613

[b40] Du C. *et al.* Hypoxia-inducible MiR-182 promotes angiogenesis by targeting RASA1 in hepatocellular carcinoma. J Exp Clin Cancer Res 34, 67 (2015). 26126858 10.1186/s13046-015-0182-1 PMC4493986

[b41] Hu P. *et al.* Minimally invasive aortic banding in mice: effects of altered cardiomyocyte insulin signaling during pressure overload. Am J Physiol Heart Circ Physiol 285, H1261–1269 (2003). 12738623 10.1152/ajpheart.00108.2003

[b42] Edgar R., Domrachev M. & Lash A. E. Gene Expression Omnibus: NCBI gene expression and hybridization array data repository. Nucleic Acids Res 30, 207–210 (2002). 11752295 10.1093/nar/30.1.207 PMC99122

[b43] Tirziu D. *et al.* Endothelial nuclear factor-kappaB-dependent regulation of arteriogenesis and branching. Circulation 126, 2589–2600 (2012). 23091063 10.1161/CIRCULATIONAHA.112.119321 PMC3514045

