# Supplementary material for: miR-182 Modulates Myocardial Hypertrophic Response Induced by Angiogenesis in Heart
Source: Sci Rep. 2016 Feb 18;6:21228. doi: 10.1038/srep21228 (PMC4758045; doi:10.1038/srep21228)
Supplement: Supplementary Information [file srep21228-s1.pdf]

SUPPLEMENTARY DATA

miR-182 Modulates Myocardial Hypertrophic Response Induced by Angiogenesis in Heart

Na Li, Cheol Hwangbo, Irina M. Jaba, Jiasheng Zhang, Irinna Papangeli, Jinah Han, Nicole Mikush, Bruno Larrivée, Anne Eichmann, Hyung J. Chun, Lawrence H. Young, Daniela Tirziu

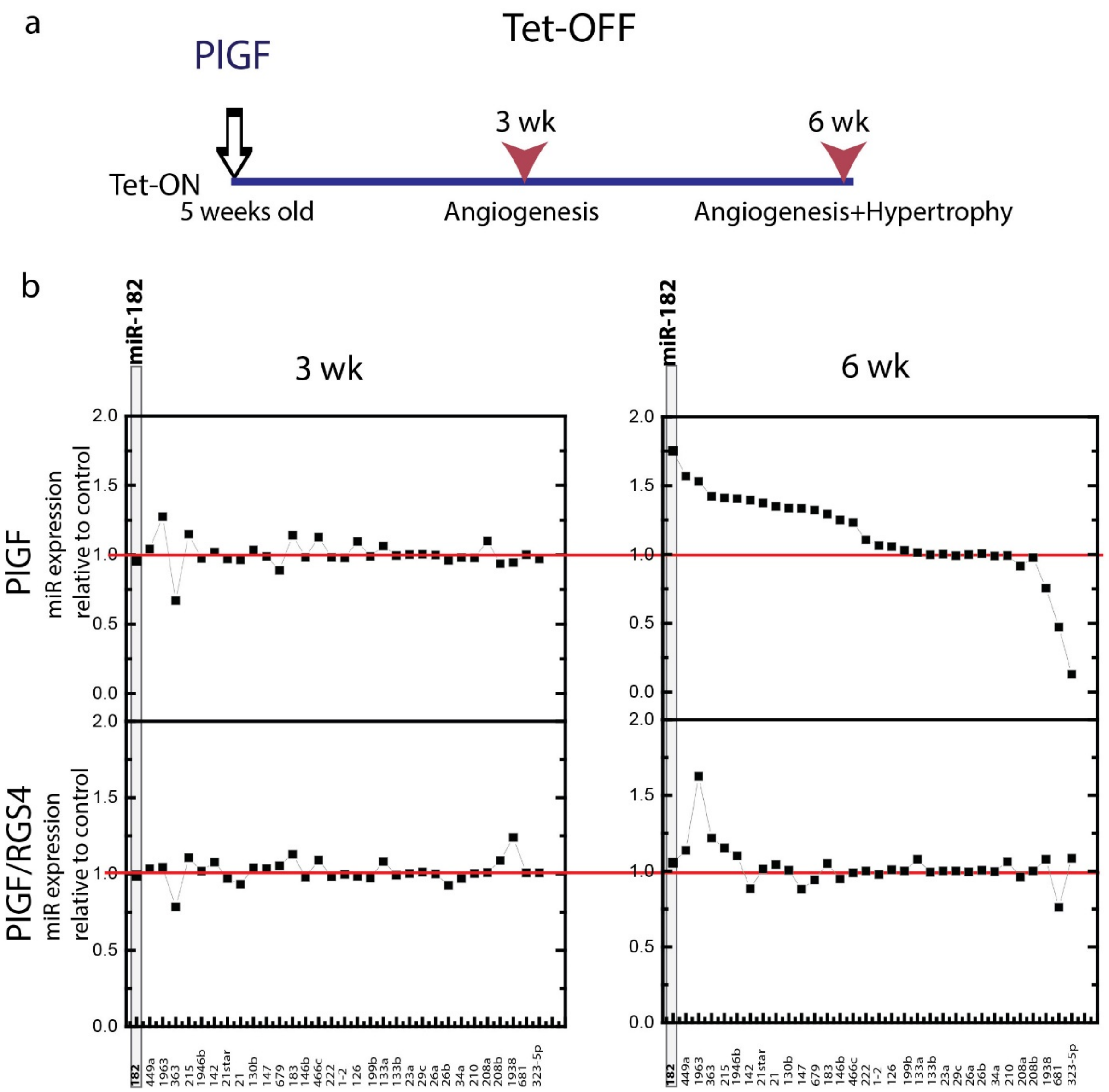

Supplementary Figure S1. (**a**) Schematic representation of the cardiac specific inducible Tet-OFF mouse model of PlGF expression. PlGF transgene expression is induced in 5 weeks old mouse heart, in the absence of docycycline (Tet-OFF) for up to 6 weeks. We previously described that angiogenesis determined after 3 weeks of PlGF stimulation preceded myocardial hypertrophy that was apparent after 6 weeks of angiogenesis.[1] (**b**) Comparative analysis of miRNA arrays (GeneChip miRNA 2.0 array, Affimetrix). Representative expression profiles of 32 microRNAs in hearts of PlGF mice (angiogenesis with the hypertrophic response) and PlGF/RGS4 mice (angiogenesis with the inhibition of hypertrophic response) relative to controls, after 3 and 6 weeks of transgene induction. Note the upregulation of miR-182 expression in PlGF mice at 6 weeks, compared with PlGF/RGS4 and control mice. No changes in expression for miRNAs previously reported as being associated with myocardial hypertrophy and heart failure such as: miR-1, miR-133a/b, miR-199b, miR-23a, miR-29c, miR-26a/b, miR-222, miR-34a, miR-210, miR-208a/b.[2-7]

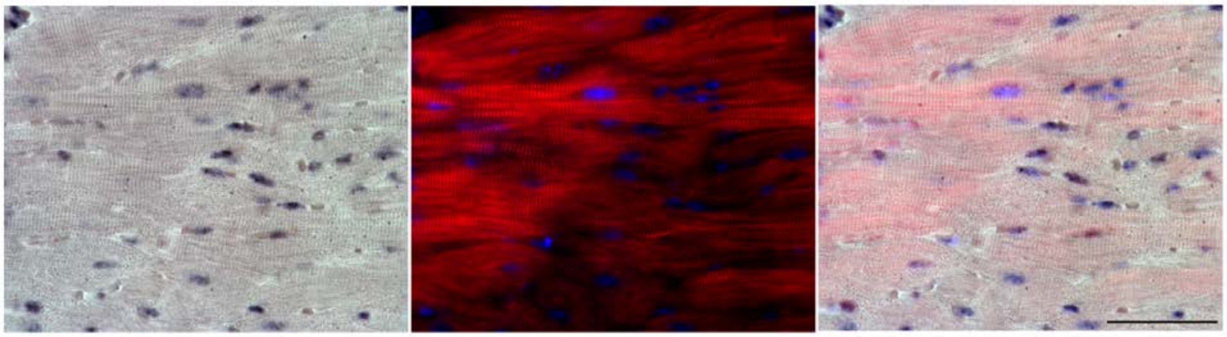

Supplementary Figure S2. In situ hybridization of LV myocardium sections with DIG labeled LNA mmu-miR-182 detection probe co-stained with an anti-cardiac troponin T antibody (cTnT) and DAPI.

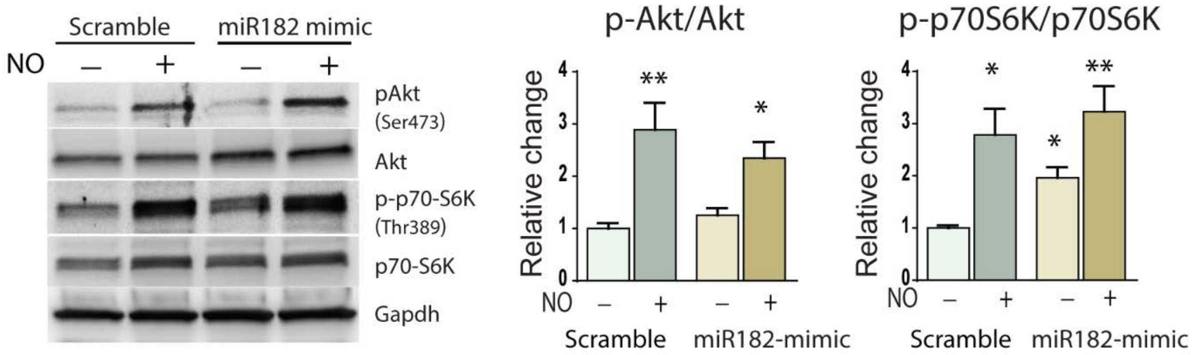

Supplementary Figure S3. miR-182 mimic treatment promotes p70-S6K^{Thr389} phosphorylation in NRCs. n=4/group, *P<0.05;**P<0.001, compared to control scramble.

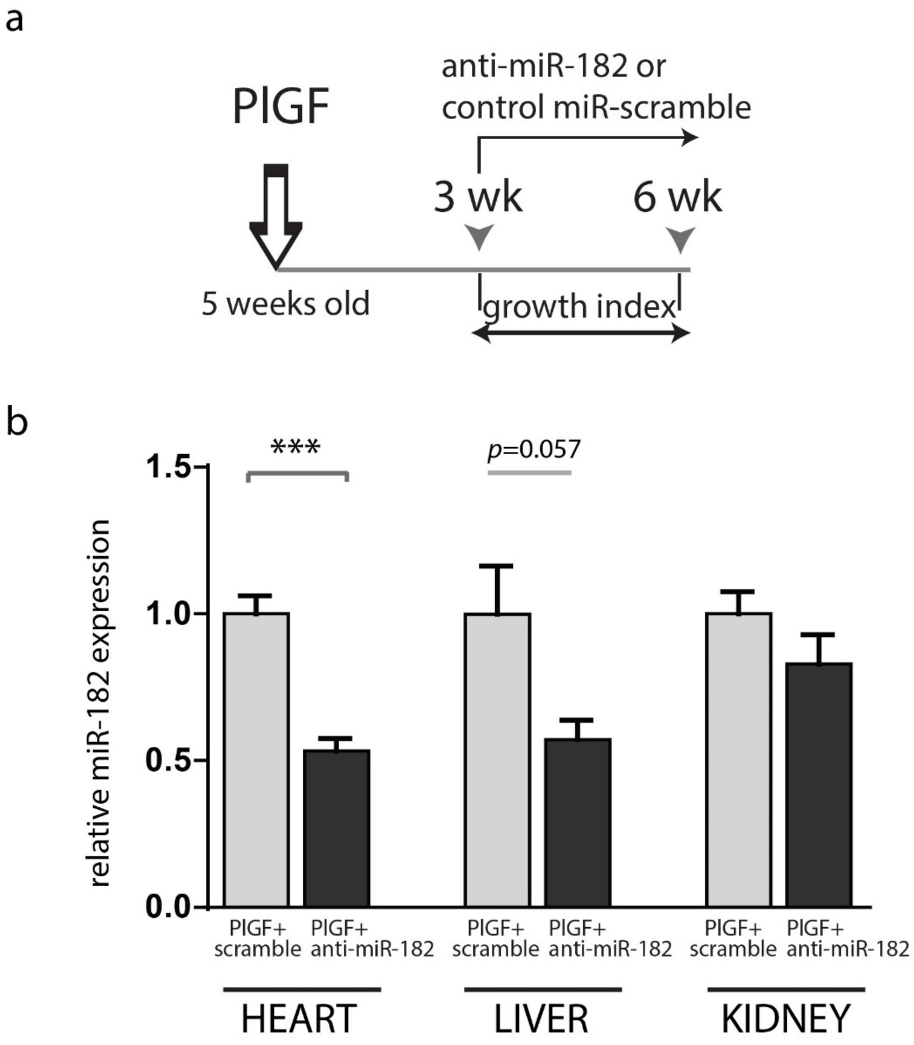

Supplementary Figure S4. Treatment of PlGF mice with anti-miR182 and control miR-scramble. (**a**). Representation of the experimental design. PlGF mice were treated with anti-miR-182 or control miR-scramble in the hypertrophic phase of angiogenic stimulation (the last 3 weeks of the 6 week period). The growth index represents the ratio of echocardiographic indicators of LV hypertrophy after treatment to measurements prior treatment. (**b**). Relative expression of miR-182 in heart, liver and kidney after the treatment with anti-miR-182. n=5 (PlGF+miR-scramble); 6 (PlGF+anti-miR-182). ****P*<0.001

Supplementary Table S1. Potential miR-182 targets identified in comparative mRNA profiling of LV samples from PlGF and PlGF/RGS4 mice vs. control mice and analysed using miRWalk database on predicted and validated microRNA targets.

		Fold-Change	p-value	Fold-Change	p-value	
Transcript Cluster ID	Gene Symbol	PlGF vs. CTRL	PlGF vs. CTRL	PlGF/RGS4 vs. CTRL	PlGF/RGS4 vs. CTRL	mRNA_assignment
10517600	**Pink1**	-1.5367	0.08357	-1.02285	0.90405	NM_026880 // RefSeq // Mus musculus PTEN induced putative kinase 1 (Pink1), nucl
10579199	**Slc25a42**	-1.5335	0.08909	1.03448	0.85926	NM_001007570 // RefSeq // Mus musculus solute carrier family 25, member 42 (Slc2
10409737	**Agtpbp1**	-1.5027	0.02089	-1.09877	0.42921	NM_023328 // RefSeq // Mus musculus ATP/GTP binding protein 1 (Agtpbp1), transcr
10368888	**Foxo3**	-1.4679	0.01582	-1.26108	0.26002	NM_019740 // RefSeq // Mus musculus forkhead box O3 (Foxo3), mRNA. // chr10 // 1
10497773	**Mccc1**	-1.4439	0.05990	-1.05203	0.72371	NM_023644 // RefSeq // Mus musculus methylcrotonoyl-Coenzyme A carboxylase 1 (al
10368356	**Akap7**	-1.4276	0.05218	-1.07495	0.58986	NM_018747 // RefSeq // Mus musculus A kinase (PRKA) anchor protein 7 (Akap7), mR
10432190	**Adcy6**	-1.3065	0.03496	-1.03071	0.73133	NM_007405 // RefSeq // Mus musculus adenylate cyclase 6 (Adcy6), mRNA. // chr15
10460400	**Pcx**	-1.3013	0.07291	1.05947	0.60249	NM_001162946 // RefSeq // Mus musculus pyruvate carboxylase (Pcx), nuclear gene
10553015	**Bcat2**	-1.2921	0.05522	-1.00185	0.98474	NM_009737 // RefSeq // Mus musculus branched chain aminotransferase 2, mitochond

References

1. Jaba, I. M. *et al.* NO triggers RGS4 degradation to coordinate angiogenesis and cardiomyocyte growth. *J Clin Invest* **123**, 1718-1731 (2013).
2. Care, A. *et al.* MicroRNA-133 controls cardiac hypertrophy. *Nat Med* **13**, 613-618 (2007).
3. da Costa Martins, P. A. *et al.* MicroRNA-199b targets the nuclear kinase Dyrk1a in an auto-amplification loop promoting calcineurin/NFAT signalling. *Nat Cell Biol* **12**, 1220-1227 (2010).
4. Ikeda, S. *et al.* MicroRNA-1 negatively regulates expression of the hypertrophy-associated calmodulin and Mef2a genes. *Mol Cell Biol* **29**, 2193-2204 (2009).
5. Sayed, D., Hong, C., Chen, I. Y., Lypowy, J. & Abdellatif, M. MicroRNAs play an essential role in the development of cardiac hypertrophy. *Circ Res* **100**, 416-424 (2007).
6. van Rooij, E., Marshall, W. S. & Olson, E. N. Toward microRNA-based therapeutics for heart disease: the sense in antisense. *Circ Res* **103**, 919-928 (2008).
7. Wang, K. *et al.* Cardiac Hypertrophy Is Positively Regulated by MicroRNA miR-23a. *J Biol Chem* **287**, 589-599 (2012).
